# Association between global leukocyte DNA methylation and cardiovascular risk in postmenopausal women

**DOI:** 10.1186/s12881-016-0335-x

**Published:** 2016-10-10

**Authors:** Ramon Bossardi Ramos, Vitor Fabris, Sheila Bunecker Lecke, Maria Augusta Maturana, Poli Mara Spritzer

**Affiliations:** 1 Gynecological Endocrinology Unit, Division of Endocrinology, Hospital de Clínicas de Porto Alegre, Rua Ramiro Barcelos, 2350, Porto Alegre, RS 90035-003 Brazil; 2 Department of Diagnostic Methods, Universidade Federal de Ciências da Saúde de Porto Alegre, Rua Sarmento Leite, 245, Porto Alegre, RS 90050-170 Brazil; 3 Laboratory of Molecular Endocrinology, Department of Physiology, Universidade Federal do Rio Grande do Sul, Rua Ramiro Barcelos, 2350, Porto Alegre, RS 90035-003 Brazil; 4 Present addresses: Institute of Cardiology of Rio Grande do Sul, Cardiology University Foundation, Avenida Princesa Isabel, 395, Porto Alegre, RS 90040-371 Brazil; 5 Unisinos University, Av. Unisinos, 950, São Leopoldo, RS 93022-000 Brazil

**Keywords:** Epigenetics, 5-methylcytosine, Aging, Post-menopause, Cardiovascular diseases

## Abstract

**Background:**

Genetic studies to date have not provided satisfactory evidence regarding risk polymorphisms for cardiovascular disease (CVD). Conversely, epigenetic mechanisms, including DNA methylation, seem to influence the risk of CVD and related conditions. Because postmenopausal women experience an increase in CVD, we set out to determine whether global DNA methylation was associated with cardiovascular risk in this population.

**Methods:**

In this cross sectional study carried out in a university hospital, 90 postmenopausal women without prior CVD diagnosis (55.5 ± 4.9 years, 5.8 [3.0–10.0] years since menopause) were enrolled. DNA was extracted from peripheral leukocytes and global DNA methylation levels were obtained with an ELISA kit. Cardiovascular risk was estimated by the Framingham General Cardiovascular Risk Score (10-year risk) (FRS). Clinical and laboratory variables were assessed. Patients were stratified into two CVD risk groups: low (FRS: <10 %, *n* = 69) and intermediate/high risk (FRS ≥10 %, *n* = 21).

**Results:**

Age, time since menopause, blood pressure, total cholesterol, and LDL-c levels were higher in FRS ≥10 % group vs. FRS <10 % group. BMI, triglycerides, HDL-c, HOMA-IR, glucose and hsC-reactive protein levels were similar in the two groups. Global DNA methylation (% 5mC) in the overall sample was 26.5 % (23.6–36.9). The FRS ≥10 % group presented lower global methylation levels compared with the FRS <10 % group: 23.9 % (20.6–29.1) vs. 28.8 % (24.3–39.6), *p* = 0.02. This analysis remained significant even after adjustment for time since menopause (*p* = 0.02).

**Conclusions:**

Our results indicate that lower global DNA methylation is associated with higher cardiovascular risk in postmenopausal women.

## Background

Epidemiologic data show that postmenopausal women experience an increase in cardiovascular disease (CVD), which could be associated with both aging and changes in hormonal status [1, 2]. In this group, higher prevalence of subclinical CVD and cardiovascular (CV) risk factors has been associated with the decline in endogenous estrogen production [3–8].

Evidence indicates that CVD results from a complex interaction between environmental and genetic factors [9, 10]. Thus, the formula proposed by the Framingham Heart study to calculate CV risk takes into account common behavioral, biochemical, and environmental aspects that may contribute to the development of CVD [11, 12]. In turn, genetic studies have shown limited predictive value of polymorphisms to explain CV risk factors [13]. This suggests that other factors might be influencing the estimated variability of CV risk [14, 15].

In a context in which traditional explanations are not sufficient to account for the link between disease, environment, and genetics, epigenetics emerges as a framework to provide insights into the bases of disease [16, 17]. Epigenetics – the study of heritable changes in gene activity or function without changes in DNA sequence [18, 19] – is highly relevant to all disease processes, including CVD and its related conditions [20]. DNA methylation, a major epigenetic mechanism, is the process by which methyl groups are added to DNA, typically regulating tissue-specific gene expression, genomic imprinting, and X chromosome inactivation [21]. DNA methylation is established and maintained by a conserved family of DNA methyltransferases (DNMTs), and plays an important role in genome stability [22]. The importance of DNMTs is related to *de novo* DNA methyltransferases 3a/3b (DNMT3a/3b) synthesis, which will methylate unmethylated cytosines to induce differential patterns of methylation in the genome of early embryos. These patterns are subsequently copied from parental strands into daughter strands during DNA replication by maintenance DNMT1 [23].

Global DNA methylation may provide a broad picture of DNA methylation changes; previous studies have shown it to be one of the earliest molecular changes in the transition from a normal to a diseased cell [24, 25]. Besides, global DNA methylation has a high-throughput, is cost-effective, and provides quantitative results [26]. Global DNA methylation changes, including decreased global DNA methylation, have been associated with clinical and subclinical CVD risk components, such as atherosclerosis, hypertension, and coronary artery disease [26–30].

Therefore, the aim of the present study was to determine whether global DNA methylation is associated with CV risk in a sample of postmenopausal women with no evidence of clinical disease.

## Methods

### Patients

This cross-sectional study was carried out at the Gynecological Endocrinology Unit of Hospital de Clínicas de Porto Alegre, Brazil. Ninety postmenopausal women from a group of 97 participants described in a previous study [31] were included in the present analysis. Seven women from the original group were excluded because we were unable to detect a methylation signal in their serum samples. As previously described [31], inclusion criteria were menopause (defined as a combination of follicle-stimulating hormone [FSH] levels above 35 IU/L and last menstrual period at least 1 year before the beginning of the study), age between 45 and 65 years, and no use of hormone therapy for at least 3 months before the enrollment. Exclusion criteria were prior diagnosis of CVD, current smoking, or a diagnosis of diabetes. The local Research Ethics Committee from Hospital de Clinicas de Porto Alegre approved the study, and each participant provided written informed consent.

### Study protocol

Anthropometric measurements included body weight, height, and body mass index (BMI, calculated as the latest measured weight in kilograms divided by the height in meters squared). Blood pressure was measured twice with a 10-min interval using an automatic blood pressure monitor (HEM-742INT; Omron, Rio de Janeiro, Brazil). Participants were in a seated position, with feet on the floor and the arm supported at heart level.

CV risk was estimated by using the Framingham General Cardiovascular Risk Score (10-year risk) (FRS), which was determined, using lipids, through the online interactive risk score calculator available on the Framingham Heart Study website [32]. Participants were stratified into two groups according to FRS: <10 % (*n* = 69), representing a low risk group; and ≥10 %, representing an intermediate/high risk group (*n* = 21).

### Global DNA methylation assays

Venous blood samples were collected. Genomic DNA was extracted from peripheral leukocytes using the technique described by Miller et al. [33]. The extracted peripheral leukocyte DNA was quantified using a NanoDrop™ 1000 Spectrophotometer (Thermo Scientific, Wilmington, DE).

Global DNA methylation levels were obtained with an ELISA-based commercial kit (MDQ1, Imprint® Methylated DNA Quantification Kit, Sigma Aldrich, St. Louis, MO, USA). The MDQ1 kit is a high-throughput molecular biology kit that uses a 96-well plate format to provide accurate differential global DNA methylation. The samples were incubated with capture and detection antibodies and absorbance was read at 450 nanometers. Quantification of global DNA methylation was obtained from calculating the amount of methylated cytosines in the samples (5 mC) relative to global cytidine (5 mC + dC) in methylated control DNA (50 ng/μL). For each sample, methylation analysis was performed in duplicate (200 ng DNA each), and the average of these measurements is reported. The intra and interassay coefficient of variation (CV) were <10 %.

### Biochemical and hormone assays

Serum estradiol, insulin, high-sensitive C-reactive protein (hs-CRP), glucose and lipid profile (triglycerides, total cholesterol, and high-density lipoprotein [HDL] cholesterol) were determined in a 12-hour fasting blood sample collected between 8 and 10 a.m. and assessed by colorimetric-enzymatic methods (Siemens Advia 1800 System, Deerfield, USA) with a coefficient of variation (CV) <3.4 %. Low-density lipoprotein (LDL) cholesterol was estimated indirectly using the Friedewald formula [34]. Serum insulin levels were measured using CLIA (Siemens Centaur XP, Deerfield, USA), with a sensitivity of 0.20 μIU/mL and intra- and interassay CV of 2.0 and 4.3 % respectively. Homeostatic model assessment index (HOMA) was calculated by multiplying insulin (μIU/mL) by glucose (mmol/L) and dividing this product by 22.5, as previously described [35]. Estradiol was measured by ECLIA (Roche Diagnostics, Mannheim, Germany), with an assay sensitivity of 5.0 pg/mL and intra- and interassay CVs of 5.7 and 6.4 % respectively. Serum hs-CRP was assayed by nephelometric method (Dade Behring Marburg, Marburg, Germany). Sensitivity was 0.17 mg/L and intra and interassay CVs were 4.4 and 5.7 % respectively. For data analysis, individual results below the sensitivity of the assay were considered as equal to 0.17 mg/L.

### Statistical analysis

Descriptive data were expressed as mean ± standard deviation (SD) or median and interquartile range (IQR) (25–75 %). Variables with non-parametric distribution were transformed into logarithms for calculation, and converted back to their original form for data presentation. Student’s *t* test was used for comparisons between group means. Global DNA methylation analyses were adjusted for time since menopause (linear regression). All analyses were performed using the Statistical Package for the Social Sciences (SPSS) version 20 (SPSS Inc., Chicago, IL, USA). Findings were deemed significant at *p* <0.05.

## Results

Considering the overall sample, mean age was 55.5 ± 4.9 years and mean BMI was 27.2 ± 4.6 kg/m^2^. Table 1 shows anthropometric and metabolic data for the entire group and for each FRS group. Patients with FRS ≥10 % were older than the group with FRS <10 %. Time since menopause, blood pressure, total cholesterol, and LDL-c levels were also higher in the FRS ≥10 % group in comparison with the FRS <10 % group. Conversely, the two groups had similar BMI, estradiol, triglycerides, HDL-c, HOMA-IR, glucose and high-sensitive C-reactive protein levels.

**Table 1:** Distribution of anthropometric and metabolic variables according to Framingham Risk Score

Characteristic	Overall (*n* = 90)	FRS <10 % (*n* = 69)	FRS ≥10 % (*n* = 21)	p*
Age (years)	55.5 ± 4.9	54.4 ± 4.4	59.1 ± 4.6	0.001
Time since menopause (years)	5.8 (3.0 – 10.0)	5.0 (2.5 – 9.0)	10 (4.0 – 13.2)	0.004
BMI (kg/m^2^)	27.2 ± 4.6	26.9 ± 4.7	28.0 ± 4.3	0.34
SBP (mmHg)	127.5 ± 17.3	120.6 ± 11.2	150.8 ± 13.5	0.001
DBP (mmHg)	78.9 ± 10.1	76.4 ± 9.2	87.3 ± 8.6	0.001
Fasting glucose (mg/dL)	93.3 ± 8.9	93.6 ± 9.1	92.1 ± 8.0	0.47
Total cholesterol (mg/dL)	216.5 ± 33.8	212.3 ± 32.7	231.0 ± 34.4	0.02
LDL-c (mg/dL)	139.2 ± 28.8	135.3 ± 27.3	152.8 ± 30.2	0.01
HDL-c (mg/dL)	53.9 ± 12.7	53.4 ± 13.2	55.5 ± 10.7	0.49
Triglycerides (mg/dL)	93.0 (72.0 – 139.7)	91.0 (70.0 – 141.2)	105.5 (75.2 – 141.0)	0.87
HOMA-IR	1.92 (1.30 – 3.19)	1.9 (1.3 – 3.2)	1.9 (1.2 – 2.9)	0.68
hs-CRP (mg/L)	1.30 (0.37 – 3.66)	1.0 (0.3 – 3.3)	2.5 (0.5 – 5.5)	0.27
Estradiol (pg/mL)	20.4 (12.0 – 31.0)	20.3 (12.0 – 31.5)	22.4 (13.4 – 30.8)	0.92
Global DNA methylation (% 5 mC)	26.5 (23.6 – 36.9)			

Data are given as mean ± standard deviation or median and interquartile range (25–75 %). *p by Student’s *t* test, FRS <10 % vs. FRS ≥10 %

*BMI* body mass index, *DBP* diastolic blood pressure, *FRS* Framingham Risk Score, *HDL-c* high-density lipoprotein cholesterol, *HOMA-IR* homeostasis model assessment - insulin resistance, *hs-CRP* high-sensitive C-reactive protein, *LDL-c* low-density lipoprotein cholesterol, *SBP* systolic blood pressure

**Fig. 1:**
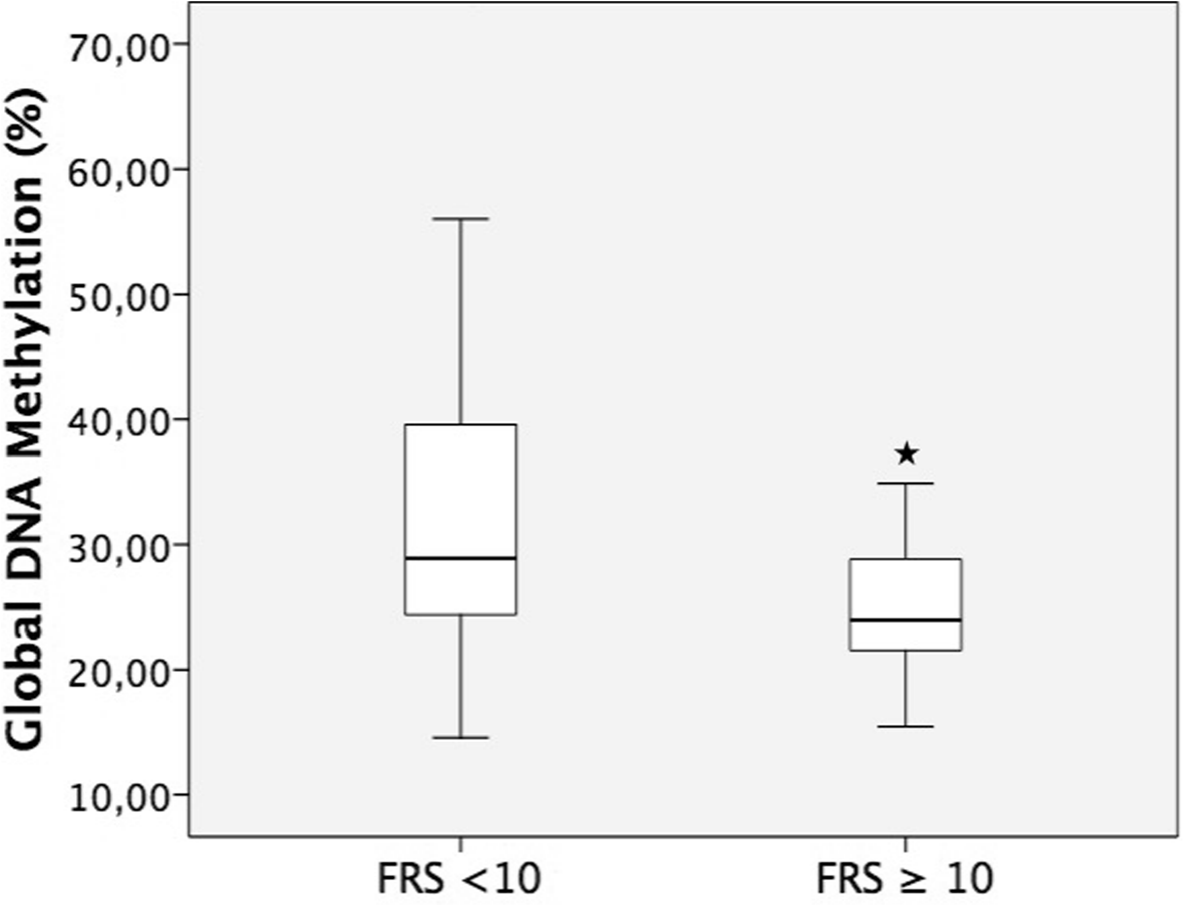
Percent global DNA methylation in FRS <10 and FRS ≥10 % groups. Values are expressed as median and 25–75 % IQR (lower and upper limit of the box); maximum and minimum values are shown by the limits of vertical lines. *p = 0.02 by Student’s *t* test

Global DNA methylation results (% 5 mC) for the overall sample and FRS groups are also presented in Table 1. The group with FRS ≥10 % had lower global methylation levels compared with the FRS <10 % group (23.9 % [20.6–29.1] vs. 28.8 % [24.3–39.6], *p* = 0.02), even after adjustment for time since menopause (*p* = 0.02) (Fig. 1).

## Discussion

In this cross-sectional study with postmenopausal women, we found that global methylation levels were lower in participants in the intermediate/high CV risk group (FRS ≥10 %) as compared to those with low CV risk (FRS <10 %). To the best of our knowledge, this is the first study to specifically examine the association between global DNA methylation and CV risk in postmenopausal women with no clinical disease.

Age and time since menopause are well-known CV risk factors [36, 37]. Indeed, decreased estrogen levels have been implicated in the progression of atherosclerosis after menopause [36, 37]. In addition, lifestyle is also linked to CV risk in the menopausal transition and postmenopausal period [38, 39]. However, in the present study, similar low circulating estrogen levels were observed in the groups with FRS <10 and FRS ≥10 % -- despite the higher age and time since menopause in the latter group. Also, the differences between these groups in methylation levels persisted after adjustment for time since menopause. This observation underscores the relevance of epigenetic mechanisms along with life style and estrogenicity for CV risk, especially in postmenopausal women.

Previous studies have associated global DNA methylation changes with clinical and subclinical CVD risk components, such as atherosclerosis, hypertension, and coronary artery disease, in different populations [26–30]. Global DNA methylation has been found to be lower in hypertensive subjects than in normotensive controls, with 5mC levels correlating with stage of hypertension [40]. Moreover, a study analyzing long interspersed nucleotide elements (LINE-1) as a surrogate marker of global DNA methylation status in blood cells found an association of lower methylation levels with cardiovascular events, such as stroke and ischemic heart disease in elderly men [41]. Another study has recently reported that lower global DNA methylation was also associated with stroke [26]. Taken together, the results of the present study and those produced by other investigators suggest that global methylation status may be regarded as a marker of CV risk in postmenopausal women. Further studies are needed in order to better understand the mechanisms underlying this association.

Animal studies have also shown that DNA methylation plays a critical role in the development of atherosclerosis and CVD. In a murine model, the absence of DNMT1 was related to increased expression of inflammatory mediators [42]. Additionally, genetically atherosclerosis-prone mice lacking apolipoprotein E showed changes in DNA methylation in peripheral blood leukocytes, which contributed to dysregulation of inflammation and promotion of atherosclerosis [43]. A modest but significant global hypomethylation status was also observed in aortic samples, and this condition preceded any histological evidence of atherosclerosis [43].

While the predominant form of methylation occurs at cytosines that are adjacent to guanine, separated by only one phosphate (CpG), in the present study we also assessed CpH sites. In this situation, a methyl group is added to a cytosine that is upstream to an adenine, thymine, or another cytosine. In fact, previous studies have shown that CpH methylation might be more common than previously appreciated [44, 45]. Moreover, CpG and CpH methylation may have distinct functions, with CpG sites methylated symmetrically and CpH sites methylated in strand-specific fashion in introns and repetitive DNA elements (SINE and LINE) [46].

Finally, we showed similar quantification of 5 mC when compared with previous studies analyzing global DNA methylation in leukocytes in different populations [47, 48]. There is a wide range of methods to evaluate methylation status. Recent advances in sequencing and microarray technology make it possible to map DNA methylation genome-wide at good resolution and in a large number of samples [49, 50]. However, these technologies may be very expensive and data processing and biological interpretation are still challenging [16]. Thus, measuring methylation levels using an ELISA assay, a well-known, simple, and reproducible technique can be helpful to guide future studies with whole-genome bisulfite sequencing.

Limitations of the present study include the cross-sectional design, which precludes conclusions regarding the direction of cause and effect. Another limitation is the relatively small sample size, which may have limited the statistical power for some analyses, such as those related to the influence of age on the observed findings. However, the effect sizes observed in our sample are similar to those reported in other populations with the same age and with established cardiovascular disease.

## Conclusion

In conclusion, our results indicate that lower global DNA methylation is associated with higher CV risk in postmenopausal women with no clinical evidence of disease. This finding should be further investigated in large-scale epidemiological studies to assess epigenetic mechanisms of CVD in this population.

## Abbreviations

BMI: Body mass index
CV: Cardiovascular
CVD: Cardiovascular disease
DBP: Diastolic blood pressure
DNMTs: DNA methyltransferases
FRS: Framingham risk score
FSH: Follicle-stimulating hormone
HDL-c: High-density lipoprotein cholesterol
HOMA-IR: Homeostasis model assessment - insulin resistance
hs-CRP: High-sensitive C-reactive protein
IQR: Interquartile range
LDL-c: Low-density lipoprotein cholesterol
LINE-1: Long interspersed nucleotide elements
SBP: Systolic blood pressure
SD: Standard deviation
